# Supplementary material for: Pneumatic Microballoons for Active Control of the Vibration-Induced Flow
Source: Micromachines (Basel). 2023 Oct 29;14(11):2010. doi: 10.3390/mi14112010 (PMC10673574; doi:10.3390/mi14112010)
Supplement: Supplementary file 1 [file micromachines-14-02010-s001.zip › supplementary_materials.pdf]

Supplementary Materials

Pneumatic microballoons for active control of the vibration induced flow

Taku Sato †, Kanji Kaneko †, Takeshi Hayakawa and Hiroaki Suzuki *

Department of Precision Mechanics, Faculty of Science and Engineering, Chuo University, Tokyo 112-8551, Japan; t_sato@nano.mech.chuo-u.ac.jp (T.S.); kaneko@nano.mech.chuo-u.ac.jp (K.K.);
hayaka-t@mech.chuo-u.ac.jp (T.H.)
* Correspondence: suzuki@mech.chuo-u.ac.jp
† These authors contributed equally to this work.

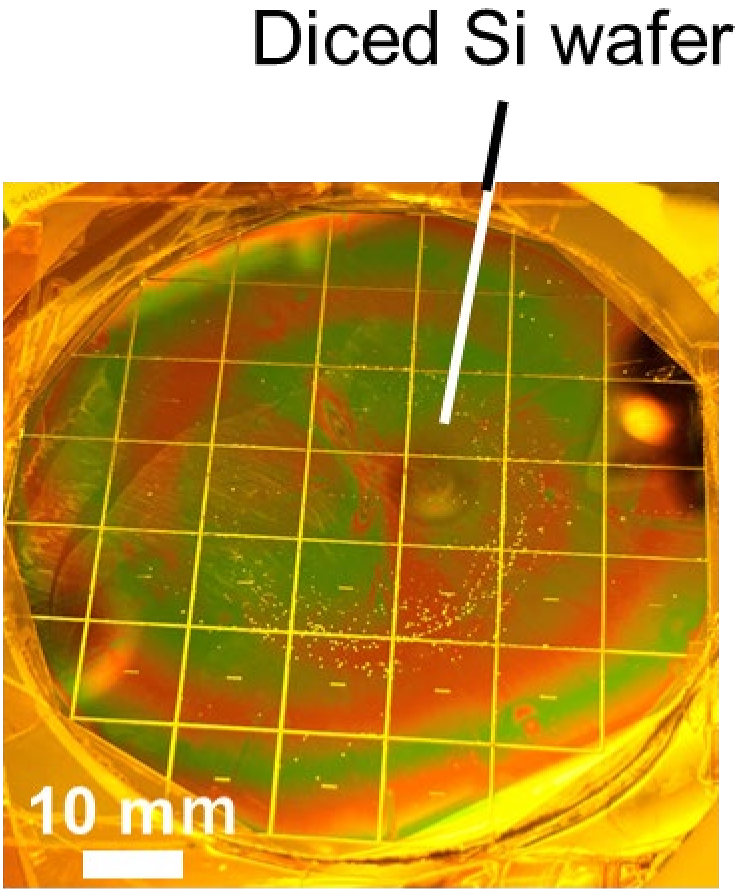

Figure S1. Actual image of the top layer of elastomer-coated Si wafer after DRIE.

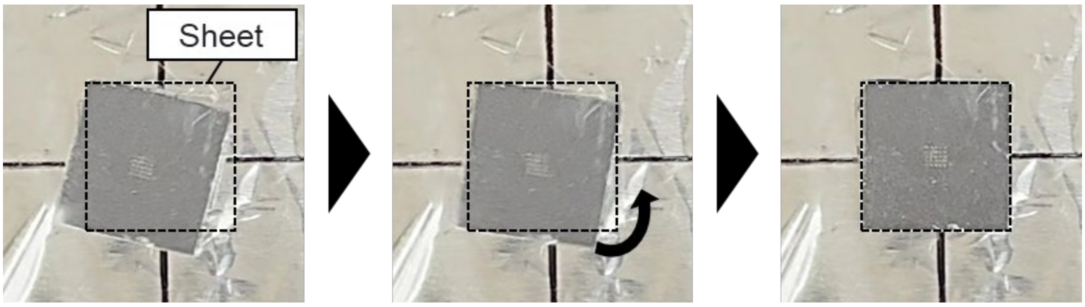

Figure S2. Alignment of Si wafer and PDMS air channel sheet via capillary action using ethanol.

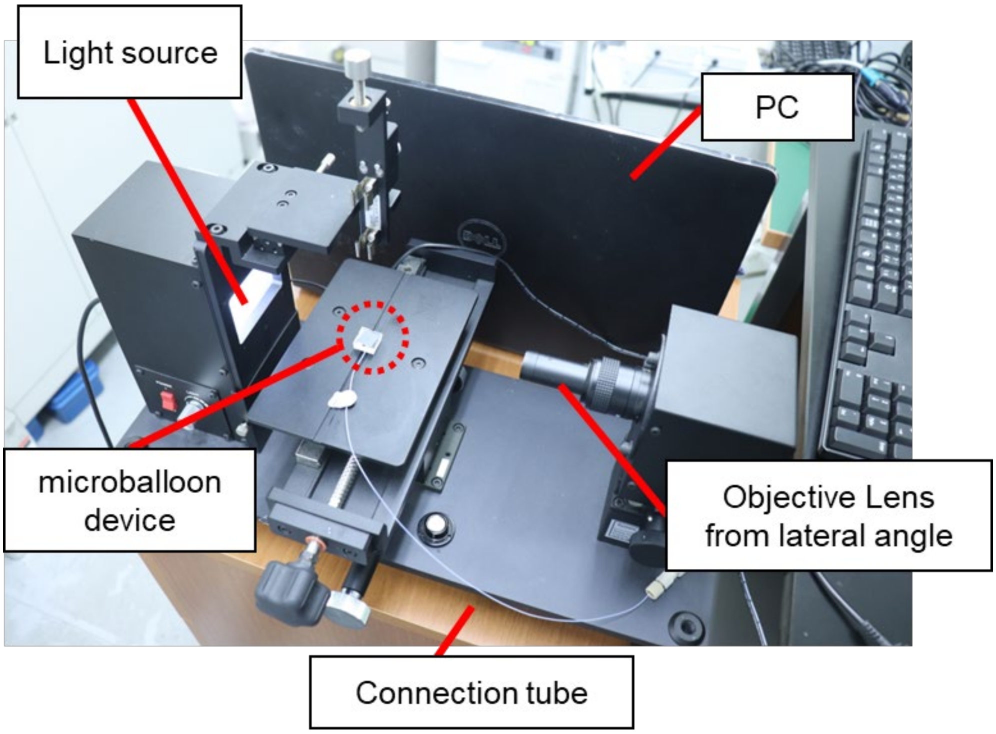

Figure S3. Experimental setup for verification of microballoon actuation via horizontal observation.

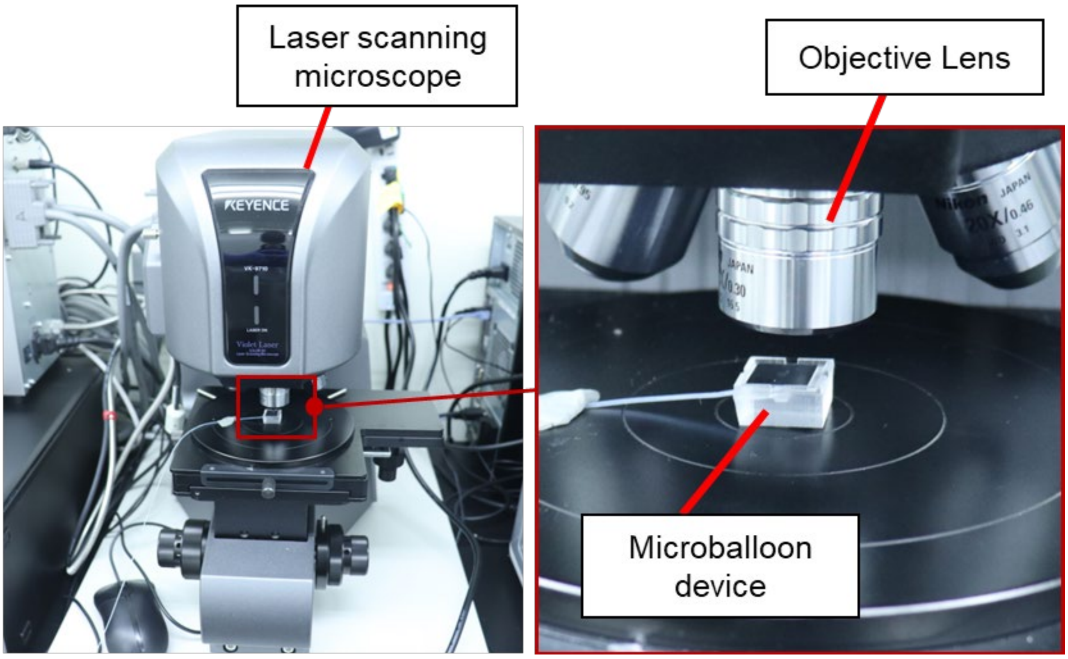

Figure S4. Experimental setup for quantitative measurement of microballoon displacement using a laser microscope.

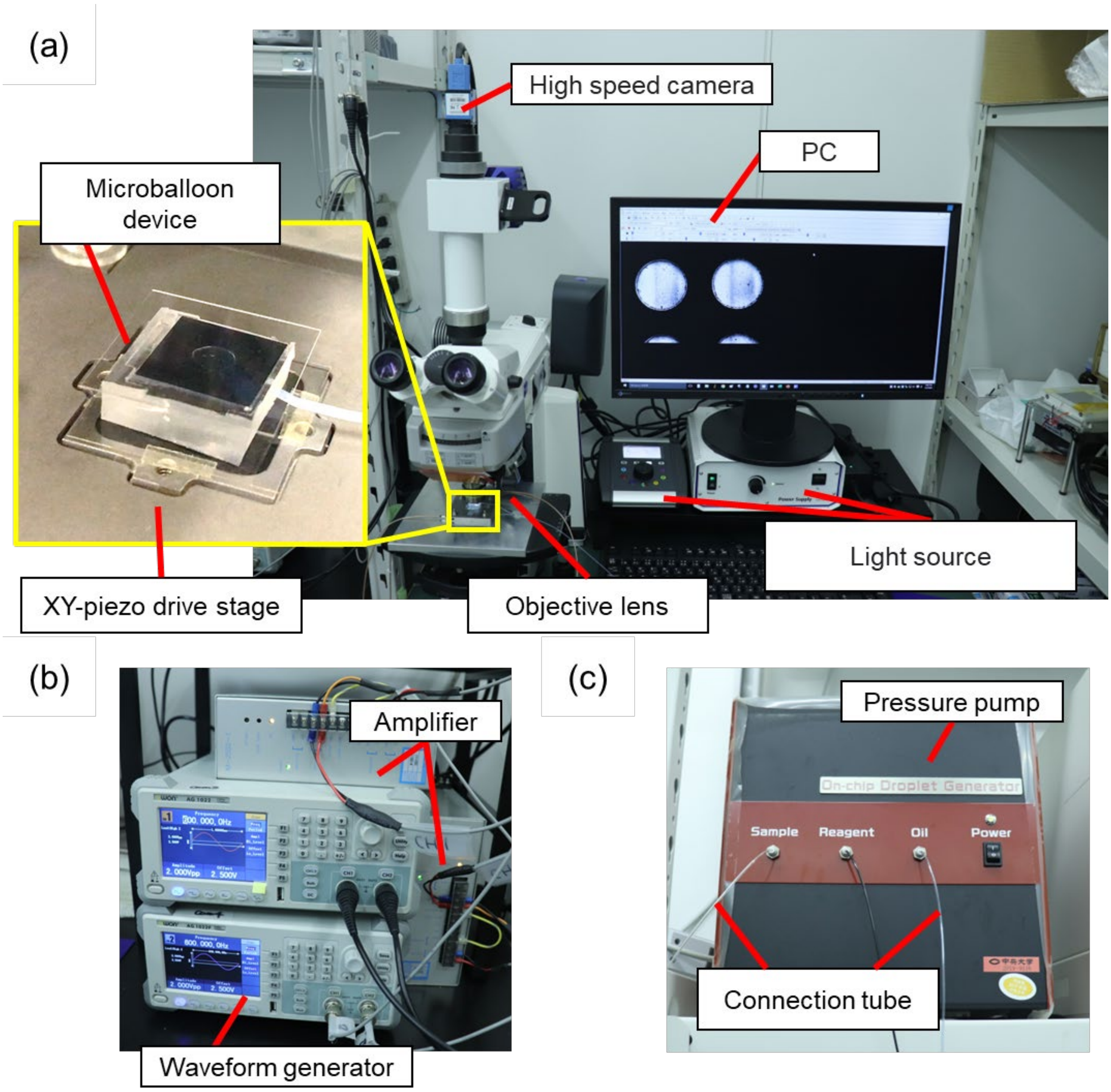

Figure S5. Experimental setup of VIF system. (a) Image acquisition system with high-speed camera for PIV measurements. (b) Input source of VIF with waveform generator and amplifier. (c) Pressure pump for actuation of microballoon.

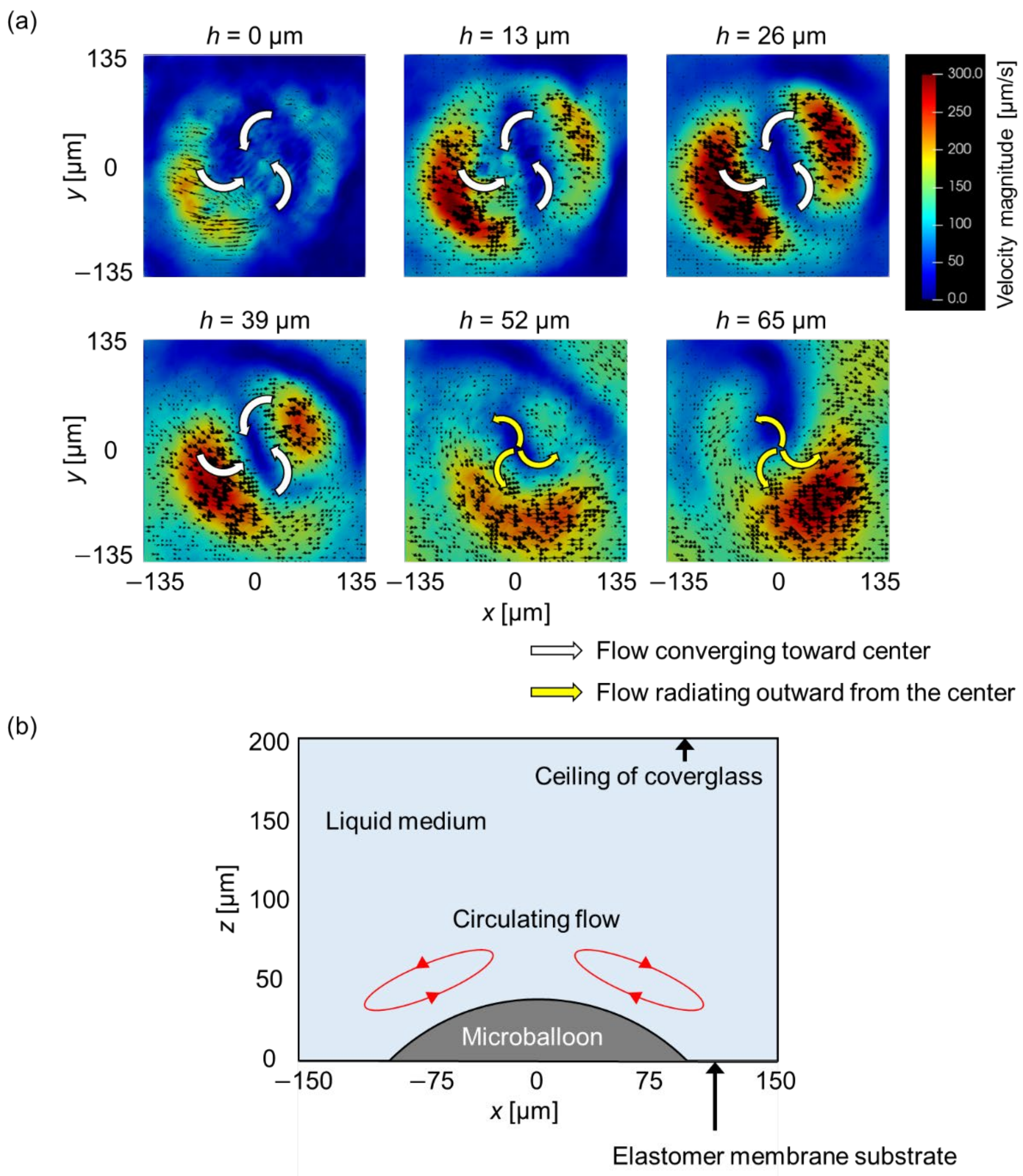

Figure S6. (a) 2D vector plots of velocity field measured using PIV at different heights. (b) schematic of three-dimensionality of flow around microballoon.

Supporting Video S1: Actual video of alignment of Si wafer and PDMS air channel sheet via capillary action using ethanol.

Supporting Video S2: Horizontal (but slightly slanted) observations of balloon actuators when 0, 10, 20, and 30 kPa was applied to all balloons. Selective actuation of actuator columns when pressure was applied to either inlet A or inlet B at 30 kPa.

Supporting Video S3: Motion of tracer particles depicted in Figure 4 in main text.

Supporting Video S4: Motion of tracer particles depicted in Figure 6 in main text.
